# E-formation à la stérilisation en situation d’isolement: solution retenue par l’armée Française

**DOI:** 10.11604/pamj.2017.26.224.9510

**Published:** 2017-04-25

**Authors:** Mederic Rouault, Marie-Audrey Vonesch, Claude Dussart

**Affiliations:** 1 HIA Legouest, Metz, France; 2 HIA Desgenettes, Lyon, France

**Keywords:** Stérilisation, militaire, e-learning, service de santé des armées, opération extérieure, Sterilization, military, e-learning, Military Health Service, external missions

## Abstract

Le service de Santé des Armées assure le soutien sanitaire des forces armées déployées en opération extérieure. Afin d'assurer la même qualité de soin sur le théâtre et en Métropole, le Service de Santé des Armées réalise la stérilisation des dispositifs médicaux réutilisables par ses propres moyens. Le pharmacien militaire en mission sur place provient de différents horizons: laboratoire de biologie médicale, recherche, ravitaillement sanitaire, pharmacie hospitalière ou encore officine pour certains réservistes. Une formation à la pratique de la stérilisation en situation d'isolement est donc nécessaire afin d'assurer une uniformité des connaissances. Notre travail s'est articulé en deux temps: détermination des besoins et des modalités de formation adaptés, puis construction de la formation proprement dite. Cette formation doit être accessible à un public dispersé géographiquement dont les niveaux d'expertise en stérilisation sont disparates. Le module « préparation opérationnelle à la stérilisation en Opération Extérieure » réalisé permet d'actualiser et d'uniformiser les connaissances des pharmaciens déployés. Il est composé de 11 sous-modules couvrant les différents aspects de la stérilisation en Opération Extérieure. Une évaluation, à l'aide de questions à choix multiples (QCM), est nécessaire pour vérifier le niveau de connaissance et de compréhension à la fin de la formation. Un taux de bonne réponse de 75% est demandé pour valider la formation. Le contenu de la formation a été approuvé par les référents nationaux en stérilisation et est d'ores et déjà disponible sur la plateforme de formation e-learning de l'Ecole du Val de Grace.

## Abstract (en)

French Army Health Service provides medical support for armed forces deployed on external missions.In order to ensure the same health care quality in the theatre and in the metropolis, the Military Health Service provides sterilization of reusable medical devices by its own means. Army pharmacists carrying out an on-site mission comes from different domains: medical biology laboratory, research, medical supplies, hospital pharmacy or yet pharmaceutical laboratory for some reservists. Training program for sterilization in isolated military operations areas is therefore necessary in order to ensure knowledge uniformity. Our study is organized in two parts: determination of the needs and of the adapted training modalities, then implementation of training itself. This training should be accessible to a geographically dispersed public whose levels of expertise in sterilization are disparate. The module «Préparation opérationnelle à la stérilisation en Opération Extérieure» carried out allows to update and standardize the knowledge of the deployed pharmacists. It is composed of 11 sub-modules covering the different aspects of sterilization during external missions. Assessment using multiple-choice questions (MCQ) is necessary to check the level of knowledge and to understanding at the end of the training. A 75% good response rate is requested to validate the training. Training content has been approved by the National referents for sterilization and it is already available on the e-learning platform of the École du Val de Grâce.

## Brève

Le Service de Santé des Armées est présent en Afrique notamment dans le cadre d'opérations extérieures. Son organisation repose sur une médicalisation-chirurgicalisation de l'avant, c'est à dire au plus près des combats, et sur le recours à l'évacuation sanitaire dès que possible pour un traitement définitif. La prise en charge sanitaire est conçue en quatre niveaux de prise en charge successifs appelés « rôles »: **le rôle 1** correspond à la relève et au conditionnement médical primaire réalisé au sein des unités de combat, c'est le poste Médicale et son médecin d'Unité; **le rôle 2** correspond au triage médico-chirurgical et à la chirurgicalisation-réanimation au plus près, c'est l'Antenne Chirurgicale; **le rôle 3** correspond au traitement des blessés sur le théâtre et à l'essentiel des évacuations sanitaires tactiques, c'est-à-dire à l'intérieur du théâtre d'opération extérieure, c'est le Groupement Médico-Chirurgicale; **le rôle 4** correspond au traitement définitif dans un Hôpital d'Instruction des Armées en France. Dès le deuxième niveau, une capacité de chirurgie est présente ainsi qu'une stérilisation. De la même manière que la qualité des soins prodigués sur le théâtre doit être équivalente à celle rencontrée en Métropole, le niveau d'assurance qualité de la stérilisation doit être le même sur place, quel que soit le niveau d'isolement que dans les Hôpitaux d'Instruction des Armées. La stérilisation est donc réalisée *in situ* par des moyens propres au Service de Santé des Armées.

En France, la stérilisation dans les établissements hospitaliers est une activité soumise à autorisation relevant de la pharmacie à usage intérieur. Le pharmacien militaire est donc l'expert désigné de la stérilisation en Opération Extérieure. Or les pharmaciens déployés sur les théâtres, pour une durée moyenne de trois mois, proviennent d'horizons très différents: laboratoires de biologie médicale, recherche, ravitaillement sanitaire, industrie, pharmacie hospitalière et même officine pour certains réservistes. Il est donc nécessaire de garantir un niveau de connaissance minimum pour chacun, sous la forme d'une formation spécifique à la stérilisation en Opération Extérieure.

L'objectif est de proposer une formation accessible à tout pharmacien partant en Opération Extérieure, quel que soit son lieu d'exercice et sa spécialité, pour remettre leurs connaissances à jour et détenir une documentation fiable et pratique. Nous présenterons ici les étapes ayant conduit à la réalisation de cette formation.

### Contexte

**Figure 1:**
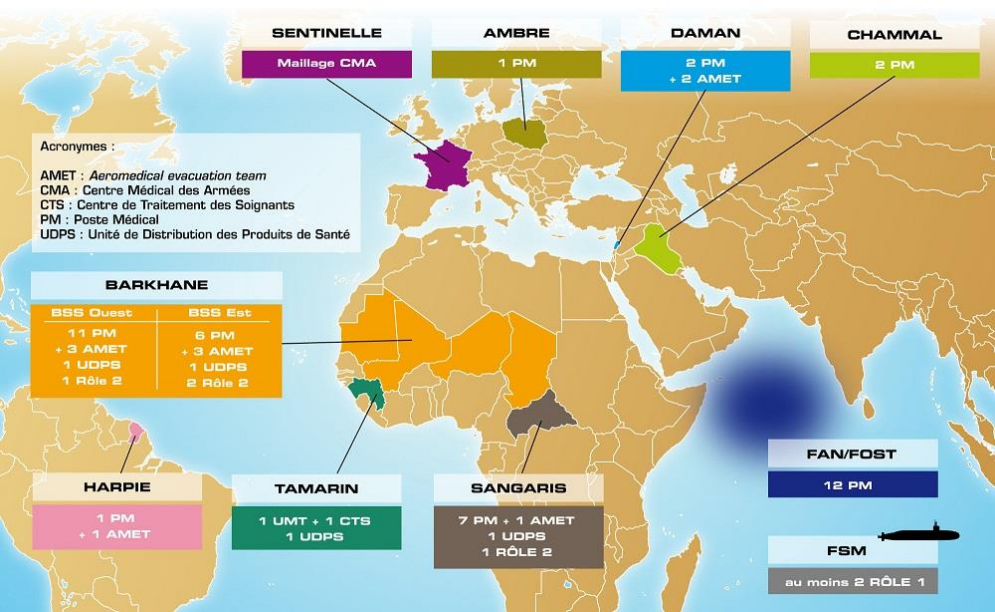
Carte des opérations extérieures en 2015

Des capacités de chirurgie du Service de Santé des Armées sont actuellement déployées dans le cadre des opérations BARKHANE dans la bande sahélo-saharienne (Mauritanie, Mali, Niger, Tchad et Burkina-Faso) et SANGARIS en Centre Afrique (**Figure 1**). En Opération Extérieure, le Service de Santé des Armées Français est organisé en « rôles »

***Rôle 1: médicalisation de l'avant:*** il correspond à la relève et au conditionnement médical primaire réalisé au sein des unités de combat. La relève comporte le ramassage des victimes et l'application de gestes d'urgence nécessaires au maintien des fonctions vitales durant l'évacuation vers l'Unité Médicale Opérationnelle de rôle 1 ou Poste Médical. Le conditionnement médical réalisé doit permettre l'évacuation sanitaire tactique de deuxième niveau vers un rôle 2. En Centre Afrique, sept postes médicaux sont déployés, et dix-sept dans la bande Sahélo-Saharienne. Il n'existe pas d'activité de stérilisation à ce niveau. Le module de chirurgie vitale (MCV) est une structure particulière armée par un médecin anesthésiste-réanimateur, un chirurgien viscéraliste, un infirmier anesthésiste et un infirmier de bloc opératoire. Il permet de déployer une capacité de chirurgie dite « de sauvetage » au plus près des forces spéciales, l'empreinte logistique est donc minimale. Il n'existe pas de capacité de stérilisation à ce niveau non plus [1].

***Rôle 2: réanimation-chirurgicalisation de l'avant:*** il correspond au triage médico-chirurgical et à la chirurgicalisation-réanimation de l'avant mis en œuvre au sein des forces. Les Unités Médicales Opérationnelles de rôle 2 peuvent être installées dans une sous structure métallo-textile ou en dur. Actuellement, il existe un rôle 2 déployé dans le cadre de l'opération SANGARIS (Centre Afrique) et trois autres dans le cadre de l'opération BARKHANE (Bande Sahélo-Saharienne). Selon les besoins, un Groupement Médico-Chirurgical est déployé (rôle 2+). Une activité de stérilisation est nécessaire au plus près du bloc opératoire. Une Unité de Dispensation des Produits de Santé dirigée par un pharmacien militaire est déployée à partir de ce niveau d'engagement. Ses missions vont de l'approvisionnement en produits de santé, au ravitaillement des Unités Médicales Opérationnelles en passant par la maintenance des matériels santé, l'entretien des dotations prépositionnées, la gestion des déchets d'activité à risque infectieux ou encore le conseil aux professionnels de santé. Il ne s'agit pas d'une pharmacie rattachée au Groupement Médico-Chirurgicale mais d'une structure distincte. La stérilisation ne fait pas partie de ses prérogatives.

***Rôle 3: traitement des blessés sur le théâtre:*** il correspond au traitement des blessés sur le théâtre et à l'essentiel des évacuations sanitaires tactiques, c'est-à-dire réalisées à l'intérieur du théâtre d'opération extérieure. Le transport entre le rôle 2 et le rôle 3 est assuré par la cellule d'évacuation médicale héliportée (Aero Medical Evacuation Team - AMET) composée d'un médecin et d'un infirmier. Le patient est alors pris en charge dans un Hôpital Médico-Chirugical, où un certain nombre de spécialités médicales et chirurgicales et d'équipements doivent être représentés pour être reconnu rôle 3 (comme la neurochirurgie). Un pharmacien dédié est déployé, avec une capacité de stérilisation. En l'absence d'Hôpital Médico-Chirugical le patient est pris en charge par l'Unité Médicale de Transit (UMT) où sont stationnés un médecin réanimateur, un médecin généraliste, un infirmier anesthésiste, deux aides-soignants et deux infirmiers. Cette équipe resserrée vise à assurer l'hospitalisation des militaires nécessitant des soins et la mise en condition avant évacuation médicale aérienne stratégique vers la France, voire vers d'autres pays d'Europe, quel que soit la gravité des patients.

***Rôle 4: traitement définitif en métropole:*** il correspond aux évacuations sanitaires stratégiques et au traitement définitif en principe sur le territoire national dans les hôpitaux d'Instruction des Armées ou dans un hôpital du même standard d'un pays allié. L'organisation et la réalisation de ces évacuations est alors placée sous la responsabilité d'équipes préconstituées, en alerte opérationnelle. Pour des pertes multiples simultanées le Module de Réanimation des Patients à Haute Elongation d'Evacuation (MORPHEE) est disponible depuis 2005. Il permet le transport de six blessés lourds ou 12 blessés légers sur plus de 7''000 km. MORPHEE est une capacité opérationnelle inscrite au dispositif de veille opérationnelle, il est intégrable en moins de 8 heures et nécessite 12 membres d'équipage et 12 personnes du secteur hospitalier ainsi que des unités médicales à vocation aéronautique. Ainsi, l'activité de stérilisation en Opération Extérieure est rendue nécessaire par l'activité chirurgicale. La sous-traitance en locale est difficilement envisageable dans le contexte d'emploi des Unités Médicales Opérationnelles, maintien de la paix en particulier. Un traitement en Métropole n'est pas envisageable, sauf dans des cas très particuliers comme l'emploi du module de chirurgie vitale. Dans ce dernier cas, l'activité de l'équipe chirurgicale est limitée par le nombre de boites stériles en sa possession.

L'activité de stérilisation en Opération Extérieure est régie par l'Instruction Ministérielle 711 modifiée par IM 2966 du 20 juin 2000 [2]. Cette instruction stipule que « Pour les troupes en campagne, en situation précaire, en opération humanitaire, en temps de guerre ou de maintien de la paix, les protocoles de stérilisation du matériel, des instruments et du linge sont identiques à ceux des services de stérilisation des établissements de soins et doivent atteindre le même niveau d'assurance qualité. » De ce fait, la réglementation applicable en Métropole, s'applique également en Opération Extérieure. La difficulté est de parvenir à atteindre et pérenniser un niveau de qualité identique à un établissement hospitalier français, avec des moyens matériels et ressources (eau) limités, dans un environnement hostile et avec des personnels renouvelés régulièrement.

Le personnel réalisant la stérilisation en Opération Extérieure n'est pas uniquement dédié à cette activité. Deux cas de figure se présentent: soit il s'agit d'une activité de stérilisation au niveau d'un rôle 2, c'est-à-dire une Antenne Chirurgicale, ou d'un dentiste, la stérilisation est alors directement sous la responsabilité du service utilisateur: soit il s'agit d'une activité de stérilisation au niveau d'un rôle 3, c'est-à-dire un Hôpital Médico-Chirurgical, la stérilisation est alors directement sous la responsabilité du service de pharmacie.

***Stérilisation rôle 2:*** accolée au bloc opératoire de la structure de niveau 2, la responsabilité de l'activité de stérilisation est dévolue à la personne la plus expérimentée puisqu'il n'y a pas de service de pharmacie. Il s'agit donc de l'infirmier de bloc opératoire le plus âgé dans le grade le plus élevé. Les infirmiers de bloc opératoire et agents de stérilisation du bloc opératoire, aides-soignants, réalisent les activités de lavage, conditionnement et stérilisation. Néanmoins, le pharmacien de l'Unité de Distribution des Produits de Santé (UDPS) reste l'interlocuteur privilégié en cas de dysfonctionnement en raison de son rôle de conseiller des professionnels de santé pour tout ce qui relève de la compétence pharmaceutique.

***Stérilisation rôle 3:*** le pharmacien de l'hôpital Médico Chirurgical est responsable de la stérilisation, mais ce sont les infirmiers de bloc opératoire et les aides-soignants du bloc opératoire qui réalisent la stérilisation. Un préparateur en pharmacie seconde le pharmacien. De plus, ces personnels proviennent d'horizons variés: le pharmacien est de préférence un pharmacien militaire exerçant dans une des pharmacies à usage intérieur des Hôpitaux d'Instruction des Armées. Cependant, tout pharmacien militaire (biologiste, chercheur, travaillant dans le ravitaillement sanitaire, réserviste ou encore occupant des fonctions de commandement) est susceptible d'être déployé sur une structure rôle 3. De plus, tous les pharmaciens hospitaliers ne font pas de stérilisation en Métropole; le préparateur en pharmacie est de préférence un préparateur exerçant dans une des pharmacies des Hôpitaux d'Instruction des Armées. Cependant un préparateur en pharmacie travaillant dans le ravitaillement sanitaire est susceptible d'être déployé sur une structure de rôle 3. De plus, aucun préparateur en pharmacie militaire ne travaille en stérilisation; les aides-soignants déployés sont le plus souvent employés dans des services de soins en Métropole ou comme brancardiers et très rarement en stérilisation.

En pratique, tout comme pour un rôle 2, ce sont les infirmiers de bloc opératoire et les aides-soignants du bloc opératoire qui réalisent l'activité de stérilisation. La validation de la charge stérilisée étant du ressort du pharmacien. Il apparait donc nécessaire de former les pharmaciens, et de manière plus générale les personnels n'occupant pas des postes en stérilisation en Métropole, à la stérilisation en situation d'isolement.

### Objectifs

Les modalités de formation sont dictées par les contraintes des apprenants. Les pharmaciens partant en Opération Extérieure sont dispersés géographiquement et leur absence pour assister à une formation en présentiel déstabiliserait l'organisation de la structure dans laquelle ils exercent. Structure qui doit déjà se préparer à une absence d'au moins trois mois d'un de ses praticiens. Une solution « à distance » est donc préférable. Cependant, la mise à disposition de documents, même s'ils sont de bonne qualité et régulièrement mis à jour ne peut pas être considéré comme une formation. Il est nécessaire de s'assurer que l'apprenant a compris et est en mesure de mettre en application ses connaissances. Une solution d'e-formation a donc été privilégiée, permettant un accès par internet à toute heure et donc une grande souplesse pour l'utilisateur [3]. Ainsi, l'apprenant suit un parcours pédagogique accompagné par un tuteur et validé par des exercices. Cette réflexion est applicable pour un grand nombre de formations du catalogue de l'Ecole du Val De Grace, qui s'est donc dotée d'un « e-campus » appelé GEDISSA (Gestion d'Enseignements à Distance et d'Informations du SSA). La solution retenue par l'Ecole du Val De Grace pour les formations en *e-learning* est la plateforme Chamilo^®^ en *open source* c'est-à-dire sans droit de licence à acquitter [4].

L'objectif est donc de proposer une formation permettant d'actualiser et d'uniformiser les connaissances en stérilisation des pharmaciens déployés en situation d'isolement. Cette formation doit être synthétique, modulable car les connaissances des pharmaciens sont hétérogènes, et surtout pragmatique. Les sujets à traiter, déterminés par une équipe de pharmaciens militaires, doivent assurer une connaissance suffisante aux pharmaciens déployés qui seront dans une situation d'isolement. Le recours à un expert en Métropole n'est pas toujours possible dans des délais compatibles avec le besoin. Le pharmacien doit ainsi être autonome.

### Contraintes techniques

La plateforme Chamilo permet de créer un parcours pédagogique à partir de présentations de type *power point* ^TM^ qui seront automatiquement transformées en ligne. L'apparence n'est que peu modifiée mais les effets de transition ou d'emphase sont supprimés. Le contenu du *power point* ^TM^ est directement accessible en ligne sans que l'apprenant ait besoin de le télécharger ou d'avoir un logiciel spécifique. Il reste possible pour l'utilisateur de télécharger tout le contenu pédagogique sur le support de son choix.

Des vidéos ou des fichiers audio peuvent être ajoutés ou associés à du contenu et il est également possible de mettre à disposition de l'apprenant des documents de référence. Idéalement, l'apprenant pourra poser des questions pratiques à un militaire expert ayant une importante expérience de la stérilisation en Opération Extérieure qui sera son tuteur. La plateforme Chamilo permet de construire une base de données de questions à choix multiples et de créer un questionnaire pour chaque sous-module. Le nombre de questions et le pourcentage de bonne réponse pour accéder au sous-module suivant est paramétrable. Ainsi, un taux de bonne réponse de 75% est demandé pour valider la formation.

### Conception du contenu pédagogique

Le contenu de cette formation a été réalisé à partir de formations internes et externes mises en place par des pharmaciens hospitaliers des Armées. Le plan détaillé des thèmes retenus a permis de mettre à jour et de consolider les formations colligées afin d'en créer une de novo sous forme de diaporama. Une fois mis en forme, les diaporamas ont été vérifiés par les référents nationaux en stérilisation. Chaque sous-module a été modifié en étroite collaboration avec les référents nationaux pour aboutir à un consensus. Afin de rendre plus pragmatique cette formation, le retour d'expérience a été privilégié à une approche plus théorique.

Le contenu pédagogique est constitué de onze diaporamas intégrés dans un parcours pédagogique sur la plateforme de formation en e-learning. Une évaluation est nécessaire pour vérifier le niveau de connaissance et de compréhension à la fin de la formation. Chaque diaporama se termine par un questionnaire (QCM) où l'apprenant doit obtenir au moins 75% de bonnes réponses pour passer au module suivant. Il reste possible d'associer une partie en présentiel pour chaque module selon les besoins. Un support physique, de type cd-rom, sera distribué aux personnels formés afin de pallier aux éventuels problèmes de réseau en situation d'isolement. Onze thèmes ont été retenus:

**Hygiène générale et risque infectieux:** contexte de la stérilisation, criticité et niveau de désinfection, les dispositifs médicaux et le risque « prion ».

**Procédés de stérilisation:** stérilisation à la vapeur d'eau saturée, autres procédés de stérilisation, stérilisation à l'oxyde d'éthylène, stérilisation à la vapeur-formaldéhyde, stérilisation au peroxyde d'hydrogène suivi d'une phase plasma, stérilisation radiation ionisantes, ce qui n'est pas de la stérilisation.

**Moyens et équipements:** présentation d'un stérilisateur à vapeur d'eau, production d'eau en stérilisation, lavage en OPEX, stérilisateurs en OPEX, module de stérilisation.

**Produits à stériliser:** usage unique et dispositifs médicaux, utilisation des dispositifs médicaux, principaux instruments chirurgicaux « universels ».

**Nettoyage et pré-désinfection:** objectifs et principes, méthodes de nettoyage et désinfection, pré-nettoyage aux ultrasons, nettoyage en machine et désinfection, nettoyage manuel et désinfection, étapes de prétraitement et nettoyage, pré-traitement, choix de la méthode de lavage, lavage en laveur-désinfecteur, lavage manuel, séchage, vérification du dispositif médical.

**Recomposition et conditionnement:** exigences générales, recomposition, matériaux et méthodes de conditionnement, papier et textile non-tissé, textile, conteneurs.

**Stérilisation:** cycles de stérilisation, résumé, introduction, phase de préchauffage d'un stérilisateur, phase de prétraitement, phase de stérilisation, phase de séchage, conditions optimales de stérilisation, exemples pratiques.

**Libération paramétrique:** aspects théoriques, aspects pratiques, test d'étanchéité au vide, test de pénétration de la vapeur, profil de la courbe de stérilisation, correspondance température/pression, durée du plateau de stérilisation, homogénéité des paramètres de stérilisation, contrôle des DMR, libération paramétrique.

**Transport, stockage et durée de conservation du matériel stérile:** locaux et équipements de stockage, zones protégées (bloc opératoire ou stérilisation), unités de soins ou Postes Médicaux, durée de conservation, valeurs guides, transport, indications à porter sur le conditionnement.

**Les contrôles en stérilisation:** maitrise de l'environnement, air, eau, surfaces, maitrise du lavage, re-qualification annuelle, tests de salissure, résumé, re-qualification annuelle, indicateurs physico-chimiques.

**Management qualité:** pourquoi un système qualité en OPEX pour la stérilisation' maitrise du processus, management qualité, responsabilité, manuel qualité, documentation, archivage, application en OPEX.

### Modalités de formation

Le choix d'une formation en e-learning a fait l'objet d'une étude SWOT (*Strength, Weakness, Opportunities, Threats*) pour Forces, Faiblesses, Opportunitées et Menaces dans la thèse du Pharmacien des Armées Elodie Ormes en 2012 [5]. Les résultats, toujours d'actualités, montraient l'intérêt d'une formation « nomade » pour les pharmaciens militaires avant départ en Opération Extérieure [6]. Plus qu'une simple mise à disposition de documents, une e-formation permet de s'affranchir des contraintes de lieu et de temps permettant également des économies sur les frais de déplacement et d'utilisations des locaux de formation. L'accès au réseau internet est une difficulté à prendre en compte mais la population visée, les pharmaciens, est en mesure d'avoir un accès sur son lieu de travail ou à défaut à domicile. L'absence d'un temps de formation en présentiel peut constituer une lacune, il est donc envisageable d'en ajouter une après la formation en ligne. Cette partie en présentiel pourrait se dérouler dans le service de stérilisation de l'Hôpital d'Instruction des Armées le plus proche. Enfin, cette formation est une réelle opportunité puisqu'aucun équivalent n'est proposé. Cette formation ne peut perdurer que si un responsable de sa mise à jour et de son animation est clairement défini.

### Contenu pédagogique

Le contenu pédagogique a été conçu pour être adapté aux besoins du pharmacien en situation d'isolement. Il a donc fallu trouver un équilibre entre l'approfondissement des connaissances et une durée de formation nécessairement limitée. Le pharmacien déployé doit être « expert » de la stérilisation, un simple rappel sur la stérilisation est très insuffisant. Les personnels de la stérilisation feront appel à lui en cas de problème, la formation doit couvrir tous les domaines de la stérilisation en Opération Extérieure et être assez précis pour apporter une expertise au pharmacien. A défaut d'apporter toutes les réponses, le pharmacien pourra stabiliser la situation en attendant un avis d'expert en France. La formation doit être suffisante pour que le pharmacien puisse déceler une situation non acceptable en stérilisation, alors même qu'il n'occupe pas de poste en stérilisation habituellement. Notre formation couvre l'ensemble de la stérilisation en situation d'isolement en onze sous modules, chacun nécessitant au moins une heure de travail. Il s'agit d'une durée à la fois faible pour un expert mais aussi très longue à intégrer dans un emploi du temps déjà bien chargé.

### Public visé

La formation a été conçue par et pour des pharmaciens, néanmoins d'autres corps de métier sont aussi en attente d'une formation de ce type. C'est le cas pour les jeunes infirmiers de bloc opératoire et les aides-soignants pratiquant la stérilisation en Opération Extérieure. Avec le transfert de compétences opéré dans les stérilisations en France, où la recomposition est principalement réalisée par les agents de stérilisation, les infirmiers de bloc opératoire sont de moins en moins présents en stérilisation. De plus, le matériel disponible en Opération Extérieure est très différent de ce qu'ils peuvent rencontrer dans leurs hôpitaux d'affectation. Ceci est d'autant plus vrai pour les plus jeunes infirmiers de bloc opératoire déployés. Les infirmiers de bloc opératoire militaires sont majoritairement formés dans le civil, il existe donc une formation d'adaptation à l'emploi lors du retour dans le milieu militaire (CADISMEX : Cours Avancé des Infirmiers Spécialisés en Opération Extérieure). Il serait possible d'insérer notre formation en e-learning entre le diplôme d'infirmier de bloc opératoire et le CADISMEX. Les apprenants pourraient ainsi avoir des bases solides pour la partie en présentiel organisée sur deux semaines. Il n'existe pas de module spécifique sur la stérilisation en OPEX dans leur formation à ce jour. Notre formation à la fois théorique et pratique est donc directement transposable pour les infirmiers de bloc opératoire. Les aides-soignants affectés en stérilisation en Opération Extérieure ne font pas de stérilisation en Métropole sauf cas exceptionnel. Il est donc nécessaire de les former avant projection. Il semble judicieux d'adapter la formation à ce public.

Afin d'assurer le même niveau de prise en charge, y compris en stérilisation, sur les théâtres d'opérations extérieures, l'uniformisation et la mise à niveau des connaissances des personnels déployés est nécessaire. C'est l'objectif de la formation réalisée sur la stérilisation en Opération Extérieure à destination des pharmaciens militaires. Il s'agit d'une formation en *e-learning* permettant de s'affranchir des contraintes de disponibilité et de lieu. Conçue autour de onze thèmes permettant de couvrir l'ensemble des activités de stérilisation en situation d'isolement, notre e-formation est validée par les experts nationaux en stérilisation militaire. La mise en place de cette formation adaptée à la pratique répond à un réel besoin sur le terrain et vient enrichir le catalogue de formation de l'Ecole du Val de Grace. Le public visé par cette formation est constitué des pharmaciens militaires déployés, mais d'autres professionnels de santé, en particulier les infirmiers de bloc opératoire qui gèrent la stérilisation au quotidien sur les théâtres, peuvent être concernés.

Ainsi, la formation « stérilisation en Opération Extérieure » est un travail qui s'inscrit dans une démarche plus globale de formation continue. Priorisant le versant opérationnel, elle s'inscrit pleinement dans les objectifs actuels du SSA. Le développement de telles formations nécessite une forte implication institutionnelle et personnelle, puisque la mise à jour en continu et son adaptation au regard des retours d'expérience sont nécessaires à sa pérennité. Enfin, notre module « stérilisation en Opération Extérieure » s'intègre dans un ensemble de 5 modules en e-learning composant la formation opérationnelle des pharmaciens militaires. Cet ensemble vise à garantir l'actualisation des connaissances des professionnels déployés notamment en Afrique.
